# The forgotten billion: bridging the global allergy care chasm through scalable, equity-driven strategies

**DOI:** 10.3389/falgy.2026.1823900

**Published:** 2026-06-10

**Authors:** Sheng-Guang Li, Lina Zhang, Jing Zhang, Ji Li, Yadan Zou, Ting Long, Ruohan Yu, Yanfeng Zhang

**Affiliations:** Department of Rheumatology and Immunology, Peking University International Hospital, Beijing, China

**Keywords:** allergy health equity, artificial intelligence in allergy, one health approach, pharmacoequity, point-of-care diagnostics, precision medicine, social determinants of health

## Abstract

**Background:**

Allergic diseases affect more than one billion people globally, yet care access is profoundly unequal. The “forgotten billion” refers to underserved populations—especially in LMICs and marginalized groups within high-income countries—who face disproportionate morbidity and preventable deaths due to gaps in diagnosis, essential medicines, immunotherapy and biologics, trained workforce, and policy prioritization.

**Objective:**

To synthesize recent (2014–2026) evidence on global burden and inequities in allergic disease care, analyze system-level gaps, review scalable innovations, present diverse case studies, and propose prioritized recommendations, monitoring frameworks, and a research and financing agenda.

**Methods:**

We conducted a structured narrative review of peer-reviewed literature and global guidance documents, focusing on burden metrics (prevalence, DALYs, mortality), access indicators, interventions (task-sharing, telemedicine, point-of-care tools, immunotherapy access, digital health, procurement/policy levers), and implementation science frameworks. Sources prioritized include WHO materials and guideline bodies (GINA, ARIA, EAACI), plus global reports and primary studies.

**Results:**

Asthma illustrates the equity chasm: in 2019 it caused approximately 21.6 million DALYs and approximately 461,000 deaths globally, with approximately 90% of burden borne in LMICs and most deaths occurring in LMICs. Systematic reviews show essential inhaled asthma medicines—especially inhaled corticosteroids (ICS) and ICS-containing combinations—are often unavailable or unaffordable in LMICs. Scalable strategies with documented impact include task-shifted care packages (Malawi), standardized primary care training (South Africa), community health worker home visiting (Boston), and public-sector access programs (Brazil), alongside national allergy strategies (Finland).

**Conclusions:**

Closing the allergy care chasm requires shifting from specialist-centric innovation to systems-first equity: universal access to essential medicines and equitable pathways to targeted therapies for severe disease, standardized primary care delivery with task-sharing, market-shaping and pricing policy reform, digitally enabled self-management designed to reduce (not widen) inequities, and integrated environmental action. Implementation must be measured with equity-sensitive frameworks and supported by durable financing aligned with UHC and NCD agendas.

## Introduction

Allergic diseases—including asthma, atopic dermatitis, allergic rhinitis, food allergy, chronic urticaria, and anaphylaxis—are both common and increasingly consequential for global health systems. EAACI's Global Atlas framing emphasizes that allergic diseases affect more than one billion people worldwide, with projections to expand further in coming decades (1). The rapid expansion of molecular diagnostics, biologics, and immunotherapy has improved outcomes for selected patients, but simultaneously highlights a profound care-access gradient: where the burden is greatest, the tools to diagnose and treat are often least available.

This paper reframes the “forgotten billion” as the people who are systematically excluded from effective allergy care—not by biology, but by the architecture of health systems and inequitable social, economic, and environmental conditions. For LMICs, this exclusion is frequently driven by constrained primary care capacity, weak chronic disease delivery models, and unaffordable or unavailable essential medicines (2). For marginalized communities in high-income countries, exclusion is shaped by structural racism, underinsurance, housing conditions, environmental injustice, and barriers to specialty care—manifesting as higher emergency utilization and mortality despite proximity to advanced health infrastructure (3).

Asthma is the most quantified allergic condition in global burden metrics and serves as the central tracer for this review. The Global Burden of Disease (GBD) estimates cited in major reviews show that asthma caused approximately 21.6 million DALYs and approximately 461,069 deaths in 2019, and that the burden is concentrated in LMICs even though prevalence is often higher in high-SDI settings (4). GINA similarly underscores that the overwhelming share of asthma deaths occurs in LMICs (2). These patterns strongly imply that the “care chasm” is not primarily a technology problem but a delivery, financing, and policy problem.

## Methods

We conducted a structured narrative review designed to support a publication-ready synthesis and action framework.

### Search strategy and sources

We prioritized evidence from January 1, 2014, through February 1, 2026, and relied on (i) peer-reviewed studies indexed in publicly accessible biomedical repositories (e.g., PubMed/PMC), (ii) major global burden and access syntheses (GBD analyses, asthma and allergy reports), and (iii) international guideline bodies and official global health sources (WHO, GINA, ARIA, EAACI) (5).

### Key concept blocks and representative search terms

We used combinations of terms related to:

- Burden and outcomes (“asthma DALYs deaths 2019”, “atopic dermatitis global burden”, “allergic rhinitis prevalence”, “food allergy prevalence”, “anaphylaxis epinephrine access”) (6),
- Access and health systems (“essential medicines availability affordability inhaled corticosteroids LMIC”, “epinephrine auto-injector availability”, “allergy workforce training”) (7),
- Scalable interventions (“task-shifting asthma trial”, “community health worker asthma home visits”, “telemedicine asthma systematic review”, “digital health rhinitis MASK-air”) (8),
- Implementation and ethics (“RE-AIM CFIR”, “algorithmic bias health care”) (9).

### Inclusion criteria

We included: (i) global/regional burden analyses (prevalence, DALYs, mortality), (ii) systematic reviews or primary studies with access metrics, (iii) trials and real-world evaluations of scalable interventions, (iv) policy and guideline documents relevant to LMIC delivery and marginalized populations, and (v) implementation science frameworks applicable to program scale-up (10).

### Exclusion criteria

We excluded small single-center case reports without scalability relevance, studies lacking clear intervention descriptions, and publications not available in English when sufficiently comparable English sources existed.

### Synthesis approach

Findings were organized along the allergy care cascade (risk/exposure → recognition/diagnosis → treatment → self-management and follow-up → prevention and system reinforcement), with explicit attention to equity mechanisms (availability, affordability, acceptability, geographic access, and quality). We then mapped interventions to delivery constraints and proposed a prioritized portfolio and evaluation indicators.

## Results

Who are the “forgotten billion”? A pragmatic definition. We define the “forgotten billion” not as a single epidemiologic cohort but as an equity-defined population: individuals with allergic diseases who experience a structural failure of access to diagnosis, essential medicines, specialist consultation when necessary, and environmental risk mitigation. This includes people in LMICs and underserved groups in high-income countries (e.g., those facing poverty, discrimination, migration-related barriers, rurality, and unsafe housing) (11).

### Global burden and inequity signals

Contemporary GBD-based analyses estimate that in 2019 there were approximately 262 million asthma cases globally and that asthma caused approximately 21.6 million DALYs and approximately 461,069 deaths; the majority of the burden is borne in LMICs, and mortality and DALYs are highest in low- and lower-middle-income categories (4). Similar GBD-based analyses estimate approximately 171 million cases of atopic dermatitis globally in 2019, with disease burden patterns that differ by SDI and age, reinforcing that “prevalence” and “harm” are not aligned when health systems differ (12). Allergic rhinitis affects more than 400 million people worldwide and is commonly underdiagnosed and undertreated, with substantial productivity loss and quality-of-life impact (13). Food allergy prevalence estimates vary by method and region; recent syntheses commonly cite high population burden (often cited as approximately 8% in children and approximately 10% in adults in some reviews), but diagnostic confirmation rates remain lower and access to gold-standard testing is uneven (14). Disparities in self-reported pediatric food allergy have also been documented in the United States (69).

**Figure 1:**
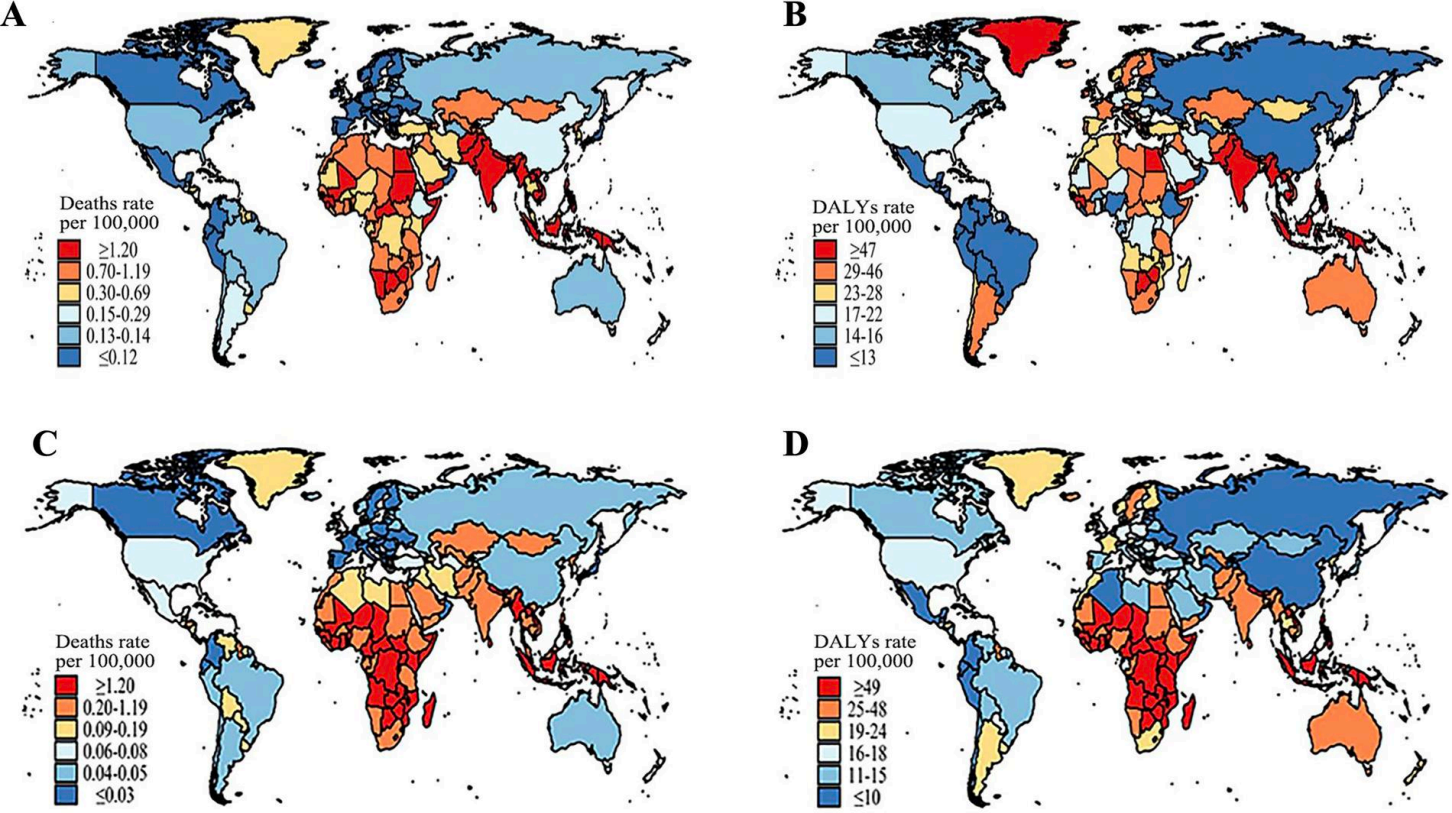
Age-standardized deaths and disability-adjusted life years (DALYs) rates (per 100,000 population) of asthma attributed to environmental risk factors in 2019, stratified by country. **(A)** Deaths attributable to smoking. **(B)** DALYs attributable to smoking. **(C)** Deaths attributable to occupational asthmagens. **(D)** DALYs attributable to occupational asthmagens. Reproduced from Zhang et al., *Environmental Health* (2024) 23:20, doi:10.1186/s12940-024-01060-8, under the Creative Commons Attribution 4.0 International License (CC BY 4.0).

These exposure-attributable patterns highlight where controller access and treatment optimization are most likely to yield the greatest mortality and disability gains, and where targeted strategies (e.g., tobacco control, workplace protection, and equitable access to effective controller therapy) are most urgent (Figure 1) (15). Having established these burden and access signals (Table 1), we next examine where the allergy/asthma care cascade breaks down—starting with gaps in recognition and diagnosis.

**Table 1:** Burden and access “signal” metrics relevant to the forgotten billion (recent evidence emphasis).

Condition (illustrative)	Recent global burden signals	Equity/access signal most relevant to underserved settings	Notes for program design
Asthma/ (1)	Approximately 262 million cases (2019); approximately 21.6 million DALYs and approximately 461,069 deaths (2019).	Most burden borne in LMICs; controller inhalers often unavailable/unaffordable.	Tracer condition for essential medicine access, task-sharing, and chronic care delivery.
Atopic dermatitis/ (16)	Approximately 171 million cases (2019).	High disability burden: access to guideline-based topical therapy and infection management can be uneven; advanced immunomodulators risk widening inequity without coverage.	Prioritize low-cost emollients/topicals, infection prevention/management, and education; scale specialty pathways selectively.
Allergic rhinitis/ (5, 9, 17)	>400 million affected globally; frequent underdiagnosis/undertreatment with major productivity loss.	Low access to accurate diagnosis and effective stepwise therapy; digital self-management may help but must address the digital divide.	Integrate rhinitis–asthma care; treat rhinitis as asthma-control determinant.
Food allergy/anaphylaxis risk/ (7, 18)	High prevalence estimates in recent syntheses; diagnosis begins with clinical history + IgE testing, with confirmatory challenges where feasible.	Severe inequities in access to EAIs across countries and within countries.	Prioritize emergency preparedness, education, and epinephrine access; harmonize school/community policies.
Cross-cutting environmental drivers/ (8)	Air pollution and climate change influence asthma/allergies and can increase severity via multiple pathways.	Disadvantaged populations have higher exposure (housing, pollution) and fewer mitigation resources.	Environmental action is part of disease control, link clinical care to legal and policy standards.

#### Diagnosis gaps

Limited tools, syndromic shortcuts, and delayed risk recognition. In many LMIC settings, asthma is underdiagnosed due to limited access to spirometry and constrained chronic care pathways; pragmatic reviews emphasize that the resulting reliance on symptom-based or syndromic diagnosis can lead to both undertreatment in LMICs and overtreatment in some high-income contexts (19). For allergic rhinitis and atopic dermatitis, diagnosis may be clinically straightforward, but correct phenotyping (e.g., comorbid asthma, severity stratification, and risk for anaphylaxis) and linkage to effective preventive therapies are often delayed (18). For food allergy, the gap is particularly pronounced because definitive diagnosis may require supervised oral food challenges, trained personnel, anaphylaxis-ready settings, and quality-assured sensitization testing. In many resource-constrained settings, the limited availability of oral food challenge capacity, standardized food allergen extracts, and reliable specific IgE testing further widens the diagnostic gap (20).

#### Treatment and access gaps

When essential medicines are “listed” but not reachable. WHO's Model List of Essential Medicines includes key asthma medicines such as inhaled budesonide and budesonide–formoterol and also includes epinephrine injection (21). Yet systematic review evidence shows that essential asthma and COPD medicines are frequently unavailable and unaffordable in LMICs, with inhaled corticosteroids (alone or in combination inhalers) particularly constrained; in many studied settings, a month's supply costs more than a day's wage for the lowest-paid government worker (22). The Global Asthma Report documents country-specific examples: in The Gambia (23), surveys found minimal availability of recommended ICS in public and private supply chains and unaffordable prices for available products; in Nigeria (24), public-sector availability of ICS has been documented as low, while oral agents that are not recommended for long-term asthma control were more available (17).

For anaphylaxis, inequities in access are especially stark. A global disparities analysis reports that EAIs are available in only a minority of countries, with absence mainly in LMICs and additional challenges of importation and high cost where they do exist (25). This creates a structural ethical problem: guideline-consistent care is not possible where first-line rescue technology is absent, despite predictable mortality risk associated with delayed epinephrine administration.

#### Workforce and training gaps

Specialist scarcity and uneven primary care competence. Allergy care is frequently specialist-dependent for diagnostic confirmation, immunotherapy, and complex cases (e.g., severe asthma phenotyping, recurrent anaphylaxis, and complex food allergy). Yet training pathways and allergy competencies are not uniformly embedded across countries; the World Allergy Organization Education and Training Committee emphasizes the need for harmonized competencies and structured training to improve allergy care delivery worldwide (26). More broadly, WHO projects a large global health workforce shortfall by 2030, concentrated in low- and lower-middle-income countries, which will constrain specialist expansion and make task-sharing models a necessity rather than a preference (27). Beyond asthma, these workforce limitations also affect oral food challenge delivery, food allergy risk assessment, anaphylaxis preparedness, and the safe implementation of immunotherapy, underscoring the need for broader allergy competencies across both primary and specialist care.

#### Policy and financing gaps

Misalignment with UHC and NCD agendas. Universal health coverage aims to ensure access to needed health services without financial hardship (28). Yet allergic diseases—and particularly asthma controller therapy—are frequently not financed as “core” packages at the level required to prevent emergency care and death. WHO's NCD action plan emphasizes targets for availability of essential NCD medicines, highlighting that access to medicines is central to NCD control, but asthma and allergy have often been underrepresented in national NCD implementation compared with other priorities (29). The financing literature on essential medicines for NCDs emphasizes that UHC financing must prioritize equitable reimbursement, procurement efficiency, and price regulation—exactly the levers that determine whether controller inhalers reach households (30).

Equity gaps within high-income countries: asthma and allergy as markers of structural injustice. In the U.S., official sources describe large racial disparities in asthma mortality and hospitalization, with markedly higher death rates among non-Hispanic Black individuals compared with non-Hispanic White individuals, including very large disparities among children. Interventions that combine clinical case management with community delivery address these inequities more directly than clinic-only models. The Boston Children's Hospital Community Asthma Initiative reported large reductions in asthma-related hospitalizations and emergency department visits among predominantly Black and Hispanic children and was launched through community-benefit and philanthropic financing with later public health support (24). To consolidate the major bottlenecks identified across settings, Table 2 summarizes the gap patterns along the allergy care cascade, their downstream consequences, and the corresponding scalable countermeasures. This gap map provides the rationale for the intervention portfolio described below, moving from diagnosis and essential medicines to delivery redesign, community models, and policy levers.

**Table 2:** Gap analysis across the allergy care cascade in LMICs and marginalized populations in high-income countries.

Domain	Common gap pattern	Consequence	Scalable countermeasure (high-level)
Recognition & diagnosis/ (1, 23, 31)	Limited spirometry; limited validated allergy testing; delayed food allergy risk stratification.	Underdiagnosis, misclassification (e.g., “recurrent bronchitis”), delayed controller therapy, preventable severe events.	Simplified algorithms + task-shared assessment; portable tools; near-patient testing with quality assurance.
Treatment access/ (1, 32, 33)	Controller inhalers and combination inhalers unaffordable/unavailable; epinephrine auto-injector scarcity.	Overreliance on relievers or oral agents; high exacerbations, ED visits, deaths.	Market-shaping procurement; essential medicine list alignment; subsidy/reimbursement reforms; in-country demand stimulation.
Workforce/ (22, 26)	Few specialists, allergy competence not embedded in general training, limited multidisciplinary teams.	Delayed escalation, limited immunotherapy capacity, insufficient education/time per patient.	Task-sharing, standardized training platforms (e.g., PACK), tele-mentoring.
Policy/financing/ (34)	Weak inclusion in UHC benefit packages; inconsistent adherence to pricing policies; low accountability for access targets.	Persistent out-of-pocket spending, catastrophic costs, inequitable outcomes.	Integrate allergy into NCD/UHC planning; use WHO pricing and procurement levers; adopt access targets and dashboards.
Environment & prevention/ (8, 35)	Poor housing, pollution exposure; limited cross-sector collaboration.	Persistent triggers, recurrent exacerbations, inequity amplifier.	One Health/environmental standards; housing remediation; school-based programs; legal protections.

#### Innovative strategies and scalable interventions

Rather than treat “innovation” as synonymous with high-cost therapeutics, we focus on innovations that can plausibly scale in resource-constrained contexts, while recognizing that biologics and other targeted therapies for severe disease raise additional equity and implementation challenges (70).

#### Task-shifting and team-based primary care delivery

Evidence supports reallocation of key asthma functions (assessment, education, inhaler technique review, trigger management, follow-up) to non-physician health workers when supported by standardized protocols and access to essential medicines. In Malawi, a randomized controlled trial of an enhanced pediatric asthma package delivered by non-physicians improved symptom control (greater improvement in cACT score) and reduced emergency healthcare attendance and school absence compared with standard physician care (23). This trial aligns with broader LMIC-focused arguments that controller inhalers and structured self-management education are the central life-saving elements, and that delivery must be adapted to workforce realities (4).

#### Standardized clinical decision support and scalable training (“protocol + practice”)

The PACK program provides an implementation blueprint: a continuously updated clinical guide paired with facility-based training using educational outreach and cascade models. Developed by the University of Cape Town (36) Lung Institute Knowledge Translation Unit, PACK was adopted as policy and scaled to thousands of facilities and tens of thousands of primary care health workers, with evidence of improved care indicators and end-user satisfaction (37). Importantly, PACK functions as a “systems intervention” more than a disease program, making it relevant to asthma/allergy integration into primary care alongside other NCDs.

#### Telemedicine and tele-mentoring for specialist reach

Telemedicine can reduce geographic barriers when integrated into workflows and designed for low-bandwidth settings; however, equity risks are substantial if connectivity and device access are limited (38). A mixed-methods systematic review of asynchronous digital health in asthma care found improvements in asthma control and reductions in hospitalizations compared with usual care, while underscoring implementation workload considerations and variable evidence quality—suggesting that telemedicine should be paired with organizational redesign rather than added on as an unfunded task (39).

#### Point-of-care and near-patient diagnostics: “good-enough” measurement that supports correct treatment

For asthma, portable spirometry, peak flow monitoring, and validated symptom/control tools can enable earlier recognition and safer step-up/step-down decisions where full pulmonary function labs are not feasible (40). For allergy, skin prick testing remains a relatively low-cost strategy but requires training, standardized extracts, and quality assurance; *in vitro* IgE diagnostics are increasingly diversified, and reviews describe how resource constraints shape test selection and the feasibility of scaling (41). Point-of-care allergen component diagnostics are an active innovation space, but scale decisions must weigh incremental diagnostic yield against affordability and supply chain realities (42).

#### Low-cost immunotherapy and disease-modifying strategies: targeted, safety-first scaling

Allergen immunotherapy (AIT) is potentially disease-modifying for selected allergic respiratory conditions and is integrated into international guidance; however, scaling in low-resource settings requires careful attention to safety, product quality, and delivery infrastructure (43). Cost-effectiveness can be context-dependent: an Indonesia-based economic evaluation in children with allergic rhinitis suggested favorable cost profiles for subcutaneous immunotherapy compared with non-immunotherapy in that setting, illustrating that “high-cost” assumptions are not universal (44). Nonetheless, AIT scale-up should not compete with universal access to essential controller inhalers and epinephrine; rather, it should be phased where safety systems and quality-assured products are available.

#### Community health worker and home-based models: shifting care to where triggers live

Home-based and community-delivered interventions address environmental triggers—pests, mold, smoke exposure—and reinforce inhaler technique and adherence, which are often the dominant determinants of preventable exacerbations (45). The Boston Community Asthma Initiative illustrates a mature model: CHW and nurse home visits reduced hospitalizations and ED visits and improved school/work attendance, providing a replicable blueprint for addressing social determinants as part of routine care (24).

#### Digital self-management in allergic multimorbidity: ARIA/MASK-air as a scalable platform concept

ARIA care pathways emphasize patient empowerment, real-life integrated care pathways, and digital mobile technology to personalize treatment and support self-management (46). The ARIA–MASK-air approach has been implemented across many countries and languages and integrates symptom monitoring, decision support, and environmental data streams, offering a model of digital support for rhinitis–asthma multimorbidity (35). For underserved populations, the crucial question is not whether apps “exist,” but whether they can be deployed with device access support, language accessibility, low-bandwidth functionality, and clinical integration.

#### Public–private partnerships and pooled procurement: market-shaping for essential inhalers and epinephrine

The Global Asthma Report explicitly argues that increasing access requires coordinated international advocacy, procurement mechanisms analogous to the Global Drug Facility, and manufacturer engagement to improve affordability of quality-assured essential asthma medicines (17). Such approaches align with WHO's emphasis on pharmaceutical pricing policies (mark-up regulation, tax exemptions, reference pricing, tendering, pooled procurement, quality-assured generics) as tools to reach essential medicines availability targets (47). In late 2025, a UN General Assembly declaration explicitly endorsed increased access to diagnosis and inhaled medicines for chronic respiratory diseases, representing a potential policy window for market-shaping and financing innovations (48).

To translate the above strategies into an implementation-ready toolkit, Table 3 summarizes a scalable intervention portfolio, including the mechanism of impact, indicative resource requirements, minimal implementation steps, and key equity risks to mitigate. We then illustrate how these components can be assembled in practice through diverse case studies, highlighting transferable design elements and common failure points.

**Table 3:** Scalable intervention portfolio with indicative costs and implementation steps.

Intervention	Mechanism of impact	Evidence signal	Indicative cost	Minimal implementation steps (scalable)	Equity risks to mitigate
Universal access to ICS-containing inhalers/ (1)	Prevents exacerbations and deaths.	Controller access is central to burden reduction in LMICs.	Moderate	EML alignment; pooled procurement; stock monitoring.	Stockouts; unequal distribution.
Task-shifted asthma education & follow-up/ (23)	Improves adherence, technique, and follow-up capacity.	Malawi RCT improved cACT and reduced emergency visits.	Low to moderate	Protocolized CHW/nurse training; clinic integration.	Under-resourced CHWs; weak supervision.
Standardized primary care decision support + training (PACK-type)/ (22)	Improves guideline-concordant care at scale.	Large-scale rollout reported in South Africa.	Moderate	Localize guide; cascade training; audit and feedback.	Protocols without medicines or supervision.
Telemedicine/asynchronous digital follow-up/ (28)	Extends specialist support and reduces travel barriers.	Improved control in selected asynchronous care models.	Moderate	Low-bandwidth platform; escalation rules; workflow redesign.	Digital divide; privacy barriers.
Epinephrine access + community anaphylaxis preparedness/ (18, 20)	Improves rescue readiness and emergency response.	Global surveys show major inequities in EAI access.	Moderate	Regulatory approval; procurement; school/community protocols.	High cost; shortages; inequitable prescribing.
Digital rhinitis–asthma self-management (ARIA/MASK-air model)/ (9, 27)	Supports symptom tracking and self-management.	Multi-country ARIA/MASK implementation experience.	Low to moderate	Validated tools; local language support; pathway integration.	Smartphone exclusion; data governance risks.
Context-adapted immunotherapy expansion (selected settings)/ (8, 44)	Provides disease modification for selected patients.	SCIT may be cost-effective in selected LMIC settings.	Higher	Quality-assured extracts; safety protocols; phased rollout.	Safety events; urban access bias.

Cost categories are indicative and context-dependent: Low (minimal new capital; uses generic medicines and CHWs), Moderate (some devices/ICT; recurring training), Higher (specialist procedures, branded technologies)

### Case studies with scalable lessons

#### Case study 1 (Brazil)

ProAR public-sector severe asthma strategy as a system lever. In Brazil, ProAR reduced admissions and costs in a low-income setting, supporting high-risk targeting, preventive inhaler access, and admissions-based accountability (49).

#### Case study 2 (South Africa)

PACK as a scalable primary care platform for asthma and beyond. In South Africa, PACK demonstrated that standardized guidance plus cascade training can support large-scale, policy-linked primary-care implementation when paired with supervision and reliable medicine supply (37).

#### Case study 3 (Malawi)

Non-physician enhanced asthma care in a randomized trial. In Malawi, a structured non-physician asthma package improved symptom control and reduced emergency visits and school absence, supporting task-sharing when training, protocols, and inhaler access are aligned (23).

#### Case study 4 (Finland)

national allergy strategy with measurable societal outcomes. Finland's national allergy program linked explicit national targets with population education and cross-sector coordination, and was associated with reduced disability, fewer hospital days, and lower costs (25).

#### Case study 5 (Boston, USA)

Community asthma delivery to reduce within-country inequities. In Boston, a community asthma model combining case management and home visits reduced hospitalizations and emergency visits among low-income children, underscoring the value of pairing clinical care with community delivery (24).

Table 4 distills these examples into transferable lessons, summarizing each program's core strategy, reported outcomes, scalable components, and common implementation failure points.

**Table 4:** Transferable lessons from selected case studies.

Case study	Core strategy	Scale and outcomes	What makes it scalable	What could fail elsewhere
Brazil ProAR/ (17)	Public severe-asthma program + free controllers.	Fewer admissions; lower family and system costs.	High-risk focus + public-system integration.	Requires stable supply and referral access.
South Africa PACK/ (22)	Standardized guide + cascade training.	Large scale-up with improved care indicators.	Platform design + iterative updates.	Fails without medicines or supervision.
Malawi pediatric RCT/ (23)	Non-physician enhanced asthma package.	Better control, fewer emergencies, less school absence.	Fits workforce constraints; outcomes are easy to track.	Needs training quality and inhaler supply.
Finland Allergy Program/ (25)	National program + population education.	Fewer hospital days; lower long-term costs.	Clear targets + long implementation horizon.	Requires strong governance and sustained funding.
Boston community asthma intervention/ (24)	CHW/nurse home visits + community design.	Fewer hospitalizations/ED visits; better attendance.	Integrated delivery + blended financing.	Needs stable funding and housing support.

## Discussion

### Actionable, prioritized recommendations

Although asthma is used here as a tracer condition because of its well-quantified burden and clear essential-medicine pathway, the same equity logic applies across allergic diseases, with condition-specific adaptations required for food allergy diagnostics, anaphylaxis preparedness, and immunotherapy delivery. The following recommendations are ordered by (i) likely near-term impact on preventable morbidity and mortality, (ii) feasibility at scale in resource-constrained systems, and (iii) equity yield.

### Priority tier 1: essential package scale-up (1–3 years)

For clinicians, programs, and ministries, the most impactful “innovation” is ensuring that evidence-based basics reach everyone.

1. Make ICS-containing inhalers universally available and affordable (through UHC coverage, procurement, and pricing policy), with explicit tracking of stockouts and household affordability (32, 33).
2. Implement task-shared asthma education and follow-up using standard protocols and supervision, leveraging nurses and CHWs where physician time is limited (23).
3. Embed standardized primary care pathways and decision support for asthma and rhinitis–asthma multimorbidity, prioritizing pragmatic tools that work without specialty infrastructure (50).
4. Treat anaphylaxis preparedness as a system obligation: ensure regulatory pathways, procurement, and community protocols for epinephrine, and measure EAI availability and affordability as equity indicators (25).

### Priority tier 2: systems and policy levers (3–7 years)

5. Align national essential medicines lists, clinical guidelines, and reimbursement lists with WHO EML and contemporary asthma guidance, updating not only what is “recommended” but what is financially protected (51).
6. Use market-shaping strategies: pooled procurement, reference pricing, tax/mark-up reforms, and quality-assured generics to reduce inhaler prices, consistent with WHO pricing policy recommendations highlighted in global asthma access analyses (52).
7. Build workforce pipelines and allergy competence via competency-based curricula, continuing education, and tele-mentoring networks—recognizing specialist growth will be slow relative to need (45).
8. Integrate environmental action into clinical programs (housing remediation referrals, clean air policies, occupational protections) using EAACI environmental and One Health frameworks as evidence-informed policy reference points (53).

### Priority tier 3: equity-first innovation (7–15 years)

9. Scale digitally enabled care pathways only when designed to reduce inequity: language access, low-bandwidth functionality, device access support, and governance for data privacy and algorithm transparency (54).
10. Expand immunotherapy access in phased, safety-first ways where quality assurance and emergency systems are mature, and where cost-effectiveness is demonstrated in context (55).

### Ethical and equity challenges

Avoiding “innovation-led inequity.” Advanced therapies and digital tools can widen gaps if they are introduced without parallel investment in essential care, workforce, and affordability. Evidence from algorithmic risk prediction demonstrates that even ostensibly “race-neutral” models can encode inequity when they use healthcare costs as proxies for need in unequal systems (68). This is particularly relevant in allergy where utilization-based models may systematically under-identify underserved patients who have less access to specialty care.

Digital divide as a structural determinant of intervention success. Telemedicine and app-based self-management can improve care access, but only where connectivity and devices are available; evidence syntheses highlight stark gaps in reliable connectivity between regions and socioeconomic groups, implying that digital-first strategies must include inclusion mechanisms (device provision, community access points, offline functionality) (56).

Safety and quality in immunotherapy and diagnostics. Scaling AIT and allergy testing requires supply-chain integrity, product standardization, and emergency response capacity. Without these, scaling can create harm and erode trust (57).

### Limitations

This review has several limitations. First, it was designed as a structured narrative review rather than a formal systematic review, and is therefore inherently more susceptible to selection bias. Second, although we applied explicit search concepts and inclusion/exclusion criteria, we prioritized policy-relevant, implementation-focused, and high-yield literature, which may have led to underrepresentation of some region-specific or disease-specific studies. Third, we did not perform a formal risk-of-bias assessment or quantitative meta-analysis. Accordingly, this manuscript should be interpreted as a policy- and implementation-oriented synthesis rather than an exhaustive evidence appraisal.

### Implementation and evaluation framework

**Figure 2:**
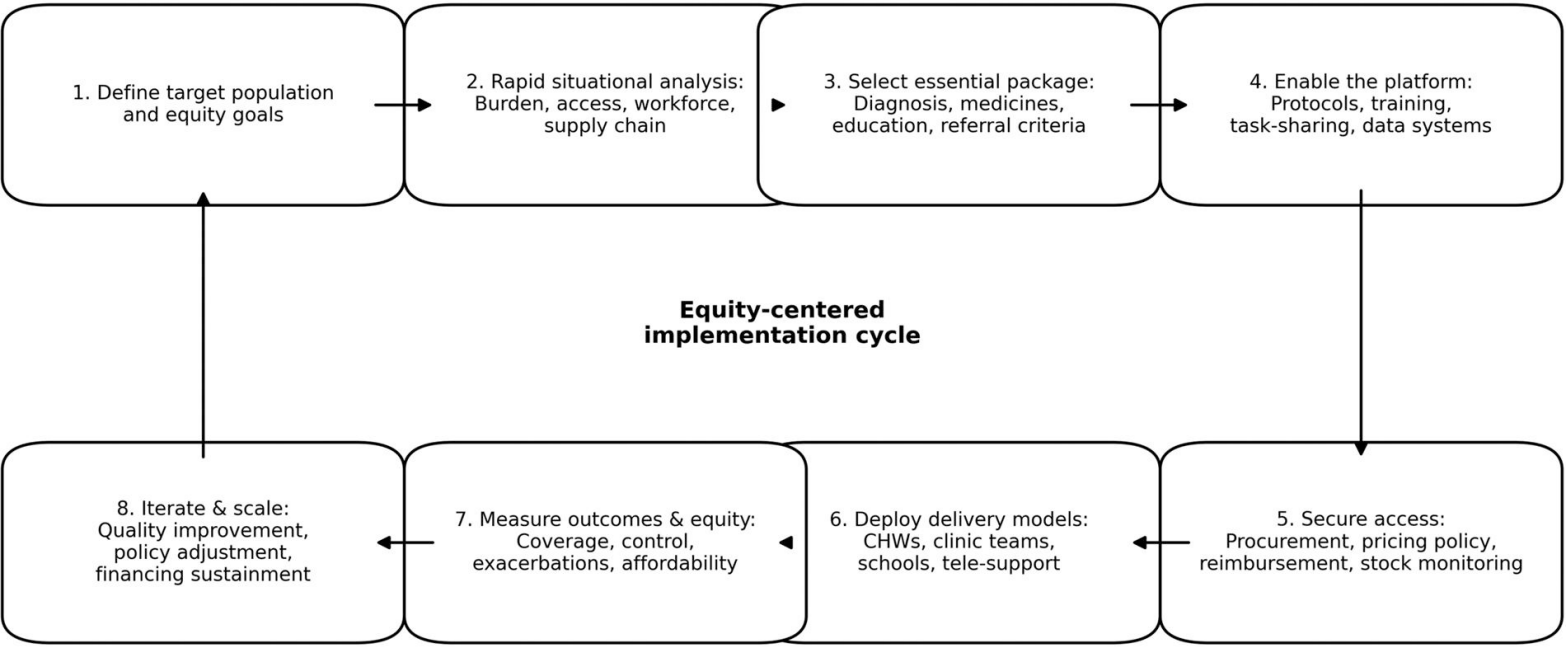
Equity-centered implementation cycle for scaling allergy/asthma care. The cycle moves from defining the target population and equity goals to rapid situational analysis, selection of an essential package, enabling the platform, securing access, deploying delivery models, measuring outcomes and equity, and iterating to scale with sustained financing and policy adjustment (1).

**Figure 3:**
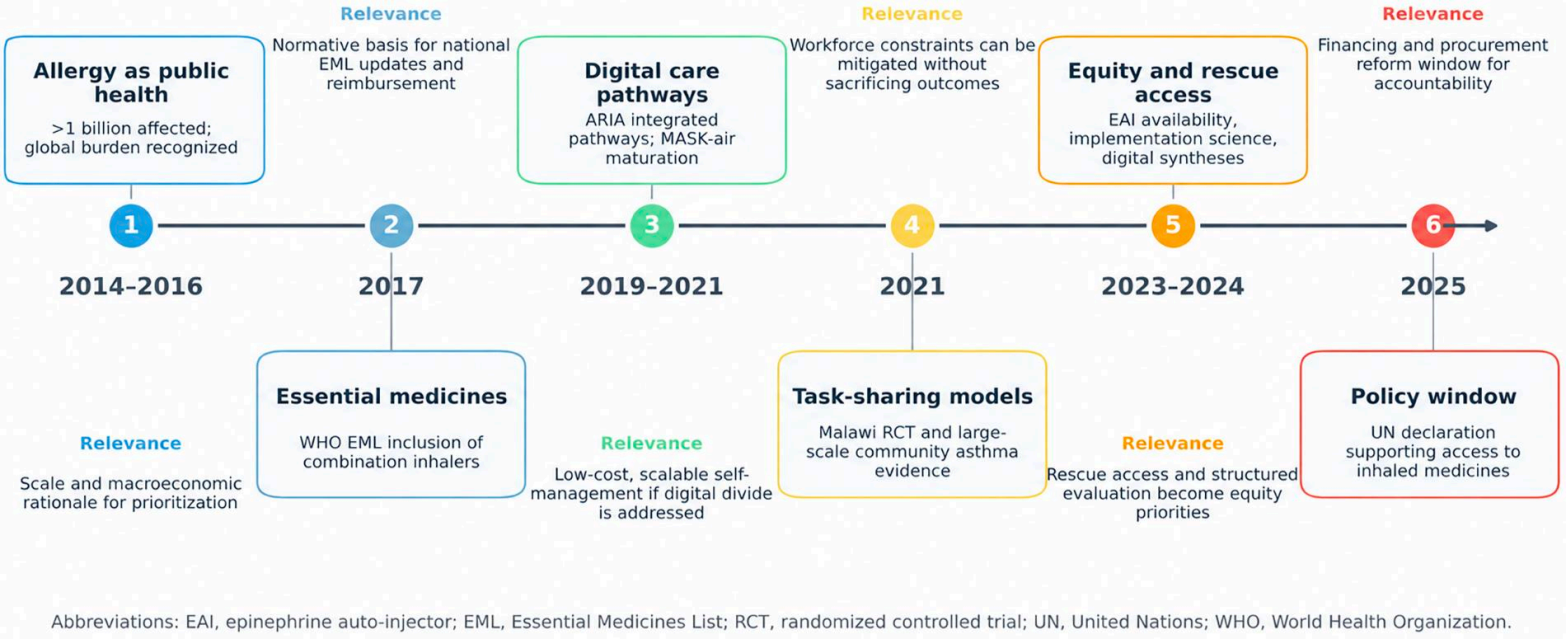
Timeline of key innovations and policy windows addressing the forgotten billion in global allergy/asthma care. The timeline highlights shifts from problem recognition to essential-medicine policy, scalable digital pathways, task-sharing delivery models, anaphylaxis rescue access, and a recent policy window supporting inhaled-medicine access.

Building on the equity-centered implementation cycle (Figure 2), we translate RE-AIM and CFIR into a pragmatic set of equity-stratified indicators that can be tracked routinely. Table 5 summarizes an equity-centered monitoring and evaluation dashboard aligned with RE-AIM (Reach, Effectiveness, Adoption, Implementation, Maintenance) and CFIR-driven context analysis (29), enabling continuous learning, quality improvement, and scale-up decisions. Figure 3 summarizes the broader sequence of innovation and policy windows that support this implementation agenda.

**Table 5:** Monitoring and evaluation framework: equity-centered program dashboard.

Domain	Indicator (example)	Equity stratifier	Data source	Program decision enabled
Reach	% of diagnosed asthma patients receiving an ICS-containing inhaler within 30 days	age, sex, income/area deprivation, rurality, ethnicity	pharmacy claims/facility registers	Identify controller-access gaps
Effectiveness	ED visits/hospitalizations per 100 patient-years	same	hospital/admin datasets	Assess reduction in acute-care burden
Adoption	% of primary care clinics trained and using standard protocols	facility type, region	training logs, audits	Target supportive supervision
Implementation quality	Stockout days for ICS/ICS–formoterol; inhaler technique competency rate	region, facility type	supply chain systems; periodic skill checks	Link procurement and training gaps
Maintenance	12–24-month retention in asthma/rhinitis care pathway	same	longitudinal cohort registers	Assess sustainment and financing needs
Safety (food allergy/anaphylaxis)	EAI availability; % high-risk patients with access to epinephrine and action plan	same	prescribing/dispensing + school policy	Detect preparedness gaps

This framework is designed to be compatible with RE-AIM (Reach, Effectiveness, Adoption, Implementation, Maintenance) and CFIR-driven context analysis (29)

## Research agenda and funding mechanisms

### Research agenda (high-yield priorities)

1. Pragmatic trials of task-sharing and simplified algorithms in LMIC primary care, including cost-effectiveness and scale-readiness (58).
2. Access science: standardized measurement of inhaler and epinephrine availability, pricing, affordability, and stockouts, linked to outcomes dashboards (59).
3. Implementation science for digital tools: comparative effectiveness of low-bandwidth telemedicine and community-supported digital self-management, with explicit equity outcomes (60).
4. Market-shaping and manufacturing strategies for essential inhalers and epinephrine in LMICs: procurement models, regulatory harmonization, and quality assurance (47).
5. Environmental intervention trials (housing, air quality, occupational exposures) that treat policy and clinical actions as coupled interventions (61).
6. Research capacity equity: increase LMIC representation in allergology research, addressing documented publication disparities and infrastructure gaps (62).

### Potential funding mechanisms

- Align allergy/asthma essential packages with UHC and NCD financing reforms supported by development partners; UHC is explicitly framed by WHO and the World Bank (63) as a core strategy to protect vulnerable populations from financial hardship while expanding access to quality services (64).
- Use blended financing: domestic budgets + catalytic philanthropy + results-based financing for measurable outcomes (controller coverage, reduced admissions). The Boston case illustrates practical blended financing (health system community benefit and philanthropic support, later public health support) (24).
- Leverage emerging global policy windows: the United Nations General Assembly (65) declaration on access to inhaled medicines provides a near-term advocacy and accountability platform for pooled procurement and access targets (48).
- Research funders should prioritize implementation science and access innovation (not only new molecules): for example, the essential-medicine access evidence base has been supported by funders including Welcomed Trust (66), highlighting that funder engagement in supply and delivery research is feasible and impactful (67).

## Conclusions

The “forgotten billion” is not defined by rare phenotypes or cutting-edge therapeutics, but by a preventable systems failure: when essential allergy and asthma care does not reach those with the highest risk of severe outcomes. Asthma's global profile makes this plain—most deaths and disability occur where controller inhalers are least available and where chronic care delivery models and trained workforce are most constrained.

Closing the global allergy care chasm requires shifting the center of gravity from specialist-centric innovation to equity-driven scale: essential medicines, task-shared delivery, standardized primary care pathways, community-based models, market-shaping procurement and pricing policy, and environmental action that reduces exposure differentials. Implementation must be measured with frameworks that reward reach and sustainment, not only efficacy in controlled settings, and must include explicit equity stratification to prevent new technologies from widening existing gaps.
